# Large deglacial shifts of the Pacific Intertropical Convergence Zone

**DOI:** 10.1038/ncomms10449

**Published:** 2016-01-22

**Authors:** A. W. Jacobel, J. F. McManus, R. F. Anderson, G. Winckler

**Affiliations:** 1 Department of Earth and Environmental Sciences, Columbia University, New York, New York 10027, USA; 2 Lamont-Doherty Earth Observatory, Palisades, New York 10964, USA

## Abstract

The position of the Intertropical Convergence Zone (ITCZ) is sensitive to changes in the balance of heat between the hemispheres which has fundamental implications for tropical hydrology and atmospheric circulation. Although the ITCZ is thought to experience the largest shifts in position during deglacial stadial events, the magnitude of shifts has proven difficult to reconstruct, in part because of a paucity of high-resolution records, particularly those including spatial components. Here we track the position of the ITCZ from 150 to 110 ka at three sites in the central equatorial Pacific at sub-millennial time resolution. Our results provide evidence of large, abrupt changes in tropical climate during the penultimate deglaciation, coincident with North Atlantic Heinrich Stadial 11 (∼136–129 ka). We identify this event both as a Northern Hemisphere increase in aeolian dust and as a shift in the mean position of the ITCZ a minimum of 4° southwards at 160° W.

## Abstract (web summary)

The extent to which the location of the Intertropical Convergence Zone (ITCZ)—a primary influence over tropical hydrology—varied in the past remains uncertain. Here, the authors use a transect of marine sediment cores to quantify latitudinal migrations of the ITCZ during the penultimate deglaciation.

## Graphical abstract

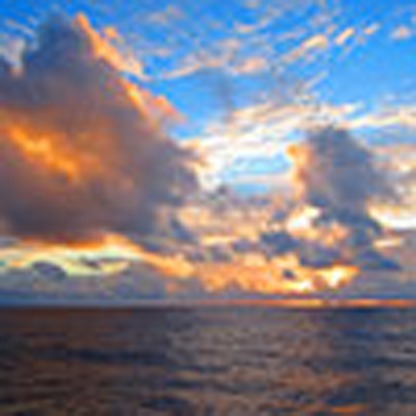

The Intertropical Convergence Zone (ITCZ) is a band of vigorous atmospheric convection and precipitation that varies position seasonally in connection with the latitude of maximum local insolation and on longer timescales in response to changes in the thermal equator^1^,^2^. This sensitivity has been reproduced in model experiments^3^,^4^ and is indicated by a variety of palaeoclimate reconstructions covering the Last Glacial Maximum, last deglaciation and Holocene^5^,^6^,^7^. Because the ITCZ monitors the hemispheric thermal balance, its past positions record the (a)symmetry of climate changes and can provide insight into the hemispheric origin of millennial and orbital climate events. As an active component of the global hydrological system, the ITCZ also propagates changes at high latitudes into the tropics by altering albedo, regional precipitation and wind fields^8^. Understanding the forcings and dynamics behind these atmospheric shifts is important for predicting future changes in the ITCZ/monsoon complex with significant implications for communities living in hydrologically sensitive environments^9^. On longer timescales, studies have suggested that the ITCZ may exert an influence on the positions of the Southern Hemisphere subtropical jet and westerly winds^10^,^11^, which it is hypothesized are an essential element of deglacial Southern Ocean upwelling^12^. Accounting for past changes in ITCZ position is thus critical to our understanding of global ocean-atmosphere dynamics, tropical hydrology and the ocean's role in regulating atmospheric pCO_2_.

As expected, given the role of ITCZ precipitation in scavenging particles from the atmosphere, previous work has found that Pacific Ocean core sites underlying the ITCZ display a latitudinal gradient in dust flux^13^ and provenance^14^,^15^. When the amount of suspended dust is the same, greater precipitation yields more dust to the underlying sediments and therefore relative dust flux can be used as a proxy for ITCZ position. Specifically, the edges of the ITCZ will have higher dust fluxes than sites within the ITCZ due to lower residual dust availability towards the interior of the ITCZ^13^. However, it is essential to recognize that a site exhibiting a temporal maximum in dust flux does not necessarily mean that the ITCZ has moved over that site, since the amount of suspended dust is not always the same above all core sites. Because the ITCZ is a barrier to cross-hemispheric dust transport^14^,^16^, variations in ITCZ latitude can force substantial changes in the provenance and amount of dust above a site. This nuance requires that we use independent dust flux data, in this case from Antarctica^17^, to inform our interpretations. Combined with our latitudinal transect and attention to relative dust flux variations between core sites, this approach allows us to differentiate between changes in atmospheric dust abundance and shifts in ITCZ position.

The central equatorial Pacific's Line Islands are well positioned to be sensitive to changes in the ITCZ because at this longitude the precipitation structure is well defined, with little deflection due to continental effects. Our records are derived from a latitudinal transect of three sediment cores: ML1208-37BB (37BB) at 7.04° N, 161.63° W and 2,798 m water depth; ML1208-31BB (31BB) at 4.68° N, 160.05° W and 2,857 m depth; and ML1208-17PC (17PC) at 0.48° N, 156.45° W and 2,926 m depth (Fig. 1). Core 37BB is located near the local modern boreal summer maximum of the ITCZ (∼7° N), and 17PC is south of the local boreal winter minimum of ∼5° N (ref. 18).

We present high-resolution records of dust flux from 150 to 110 ka at three sites in the Line Islands to constrain the ITCZ position during the penultimate deglaciation (Termination II or TII). These records place latitudinal constraints on the millennial-scale movement of the Pacific ITCZ over this time period. Our data reconstruct a large, abrupt change in tropical Pacific climate over TII, characterized by both an increase in atmospheric dust abundance and by a shift of the ITCZ of at least 4° southwards. This tropical event is coincident with Heinrich Stadial 11 (HS11). After TII, dust fluxes declined to interglacial levels and the ITCZ shifted northwards of its previous glacial position.

## Results

### Sedimentation rates

Sedimentation rates vary between cores and within cores with average rates for the interval studied (110–150 ka) of 1.1, 4.8 and 3.0 cm ka^−1^ for cores 37BB, 31BB and 17PC respectively. A higher level of confidence is placed on conclusions when the sedimentation rate is higher, notably for the deglacial interval key to our primary interpretations in this paper. Details on the development of the age models from which these sedimentation rates are derived may be found in Methods.

### Dust flux data

**Figure 2:**
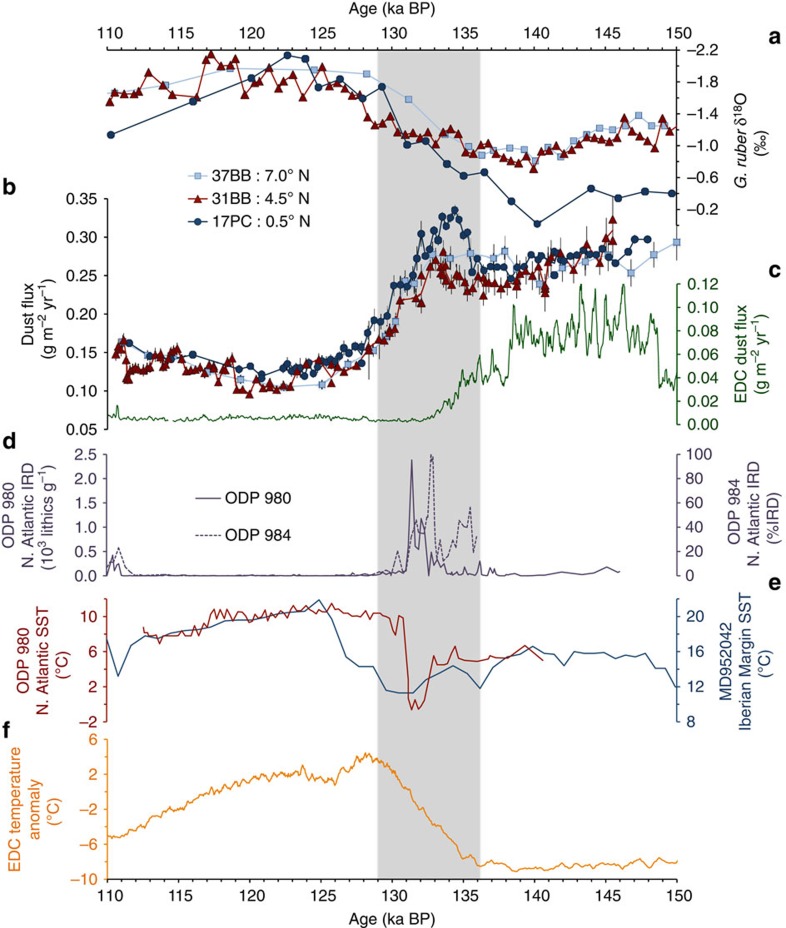
Climate of the penultimate deglaciation. Planktonic foraminifera δ^18^O stratigraphy for cores 37BB (light blue squares), 31BB (red triangles) and 17PC (navy circles)^19^ (**a**). Dust flux records from 37BB, 31BB and 17PC. Symbols as in **a**, error bars are 2 s.d. Lines connect data points and the mean value of replicates (**b**). Dust flux from Antarctic ice core EDC^17^ (75°06' S, 123° 21' E) on AICC2012 chronology^41^ (5 pt smoothed) (**c**). North Atlantic (N. Atlantic) ice-rafted debris (IRD) abundance from sites ODP 980 (55° 29' N, 14° 42' W) and ODP 984 (61° 25' N, 24° 4' W)^42^ (**d**). North Atlantic sea surface temperature (SST) proxy records from ODP 980 (ref. 29) on the timescale of Mokeddem *et al*.^42^ (red line), and MD952042 (ref. 43; navy blue line) (**e**). Antarctic EDC temperature anomaly record^44^ on AICC2012 chronology^41^ (5 pt smoothed), where the anomaly is relative to the last 1,000 years (**f**). Grey shading represents Weak Monsoon Interval II (WMI-II)^27^ correlated with HS11 in the North Atlantic. Principles underlying the interpretation of proxies in **e** and **f** are discussed in Supplementary Note 2.

Dust fluxes were reconstructed using ^230^Th_xs,0_-normalized ^232^Th fluxes (Supplementary Data 1) as described in Methods. Data for sites 37BB, 31BB and 17PC (Fig. 2b) show similar trends in time from Marine Isotope Stage (MIS) 6 through MIS5. During MIS6 (oldest part of record to ∼140 ka) all cores show high dust deposition (>0.25 g m^−2^ yr^−1^) relative to the interglacial. At the peak glacial (as marked by the maxima in oxygen isotope values; Fig. 2a) the northernmost core is the first to show a small increase in dust flux, followed by the two more southerly cores between ∼136 and 135 ka. Deglacial dust flux maxima occur in cores 31BB and 17PC between ∼133.4 and 134.5 ka, and after that values in all cores decline precipitously reaching a minimum (0.1 to 0.15 g m^−2^ yr^−1^) during the MIS5 interglacial. This interglacial dust flux value is ∼2.5 times lower than glacial values. For the remainder of the records all three cores show gradually increasing dust fluxes.

### Oxygen isotope data

Oxygen isotope data generated on the planktonic foraminifera *Globigerinoides ruber* in ML1208 cores 37BB, 31BB and 37BB (Fig. 2a) have been published elsewhere^19^, but here we provide additional interpretation of these data in the context of our inferences about MIS6-5. Planktonic oxygen isotope (δ^18^O) data record a combination of surface water conditions including ambient temperature, salinity and the global δ^18^O of sea water. These variables in turn reflect changes in sea-surface temperature, local precipitation, upwelling and global ice volume. Variations in δ^18^O may reflect either local processes or the signature of waters advected to our study sites, or a combination of both. Although we cannot parse the contributions of each of these variables to observed changes in the records, it is instructive to consider the relationship between δ^18^O data from the three cores and how it changes over time.

From 150 and 140 ka, oxygen isotope values for cores 37BB and 31BB are between −1.28 and −0.8‰, representing substantially fresher or warmer conditions than at site 17PC (−0.5 to −0.02‰). As the deglaciation begins (∼140 ka) δ^18^O values freshen and warm at all three sites in response to rising global temperatures and reduced ice volume. However, during HS11 (136–129 ka), site 17PC shows the largest change (1.07‰), indicating that freshening and/or warming was most strongly felt at the southernmost site. At the peak of MIS5e (∼120 ka), the δ^18^O recorded by foraminifera at sites 37BB, 31BB and 17PC is similar.

## Discussion

Few dust flux records exist from the central equatorial Pacific, but a low-resolution record from TT013-72PC (72PC) at 0.1° N, 139.4° W (ref. 20) provides an important comparison for our sediment cores. Data from 72PC show a similar decrease in dust flux from 145 to 118 ka, followed by an increase from 118 to 110 ka, in agreement with our results. Given the paucity of high-resolution marine dust flux records covering TII, the Antarctic EPICA Dome C (EDC) dust record is a valuable companion data set (Fig. 2c). Antarctic and equatorial dust fluxes co-vary at coarse resolution between hemispheres during the late Pleistocene ice-age cycles^20^, and in this context it is the differences between our high-resolution records and the EDC record that are the most significant. Specifically, the decline in EDC dust flux at the MIS5/6 boundary (TII at ∼134 ka; from ∼0.036 to 0.007 g m^−2^ yr^−1^) trends oppositely to the increase in dust flux observed at sites 37BB, 31BB and 17PC during HS11 (grey shading in Fig. 2). Because of the relatively high resolution of the EDC ice core and a comparable decline in dust flux in at least one other high-resolution, Southern Hemisphere record^21^, we hypothesize that the absence of a HS11 dust peak in the ice core record indicates that the observed dust peak in the central equatorial Pacific does not reflect an increase in global or Southern Hemisphere dust abundance, but rather an increase in northern hemisphere sources alone. Independent behaviour of Northern and Southern Hemisphere dust sources has been repeatedly observed for Heinrich stadials over the last glacial cycle^17^,^22^ (Supplementary Fig. 1c,d), and we propose that a similar hemispheric dust flux asymmetry characterized TII during HS11. Although no high-resolution dust records exist from the Northern Hemisphere covering HS11, we suggest that the increase in Northern Hemisphere dust that marked previous Heinrich stadials also characterized HS11 (ref. 23), especially since the relatively long duration of HS11 (ref. 24) would likely have allowed for the full expression of stadial conditions. Other indicators of stadial conditions^25^ have also identified HS11, including high ice-rafted debris (IRD) abundance in North Atlantic sediments (Fig. 2d) and cold sea-surface temperatures (Fig. 2e). While not causally related to dust fluxes, these stadial indicators are repeatedly seen to change in concert with increases in Northern Hemisphere dust abundance. Recurrent intervals of high dust flux during Heinrich stadials, in combination with the clear evidence for decreased dust abundance in the Southern Hemisphere, strongly support the idea that the dust flux increases in our central equatorial Pacific cores are due to a Northern Hemisphere dust increase.

**Figure 3:**
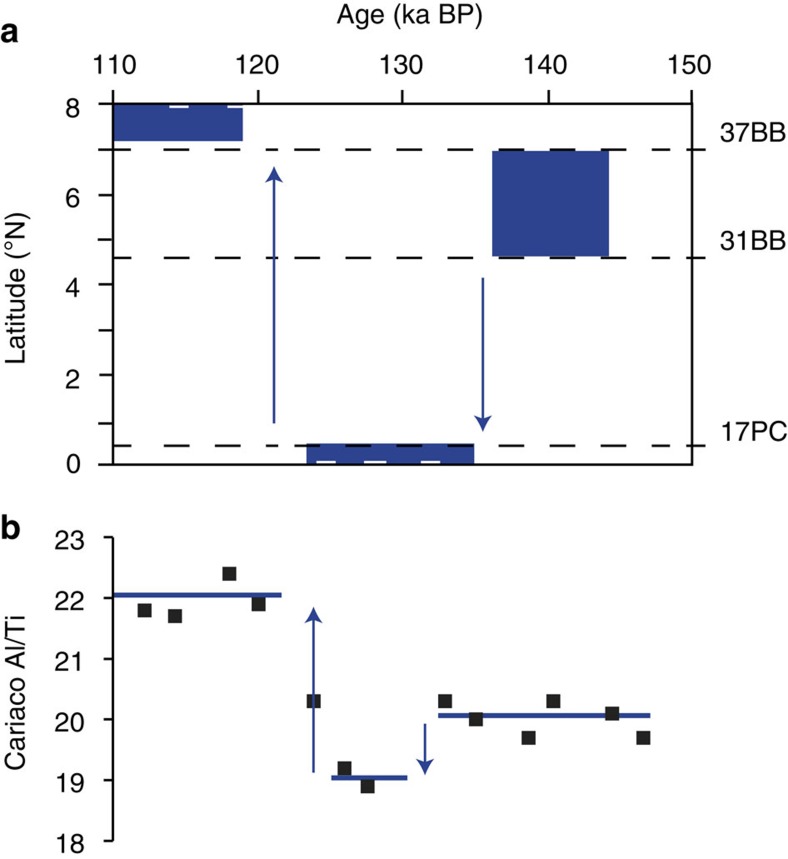
Termination II ITCZ shifts. Blue boxes indicate range of possible latitudes of the mean ITCZ position, black dashed lines denote core latitudes (**a**). Cariaco Basin Al/Ti record (black squares)^28^ with blue bars indicating estimates of the relative ITCZ position through time (**b**).

Although atmospheric dust abundance clearly varies on deglacial timescales, it cannot explain the time-variant differences in dust flux among our three sites. Instead, we attribute inter-core differences to changes in the position of the ITCZ (Fig. 3a). Our interpretations of ITCZ movement take into account our new dust flux records, planktonic oxygen isotope data from the same three sites, variations in Southern Hemisphere dust abundance as recorded in Antarctica^17^ and temperature proxy records that indicate the thermal gradient between poles (Fig. 2e, f and Supplementary Note 2).

During MIS6 (∼145 ka) dust fluxes at the three sites do not show a sufficiently clear relationship to infer confidently, on dust flux data alone, the position of the ITCZ. Instead we suggest that the similar oxygen isotope values seen at sites 37BB and 31BB indicate an ITCZ positioned approximately equidistant between the two cores. Fresher conditions at these two sites would be consistent with ITCZ-derived precipitation located directly above the sites. Colder and/or more saline conditions at 17PC are also consistent with this interpretation, as cross equatorial winds could have driven enhanced upwelling at this southernmost site as they do seasonally at present when the ITCZ is at its northernmost position^26^.

Between ∼136 and 135 ka, coincident with the onset of HS11 in the North Atlantic (grey bar Fig. 2), dust fluxes increase in 31BB and 17PC, which we interpret as indicative of a southward shift in the northern edge of the ITCZ. Because dust fluxes decrease in the Southern Hemisphere from 138.5 ka into MIS5, and because an increase in dustiness is repeatedly associated with Heinrich stadials, increasing dust fluxes in cores 31BB and 17PC indicate that they are likely receiving dust from the Northern Hemisphere. Since the ITCZ acts as a barrier to dust, the observed dust increases at 31BB and 17PC imply that the ITCZ must have been south of both of these sites. Additional evidence for an ITCZ south of 0.48° N during HS11 comes from contrasting the 24% increase in dust flux at the southernmost site (17PC), with the record from the intermediate core (31BB); this shows only a 9% increase, despite a higher sedimentation rate in core 31BB during this interval of ∼3.9 cm ka^−1^ relative to a rate of ∼2.9 cm ka^−1^ in core 17PC over the same time interval (see Methods). These increases in dust flux represent mean values for a range of data points before HS11 and during the dust flux maximum, with a maximum change in core 17PC of 32% and a minimum of 15%, and a maximum for core 31BB of 21% and a minimum of 1%. Here more efficient dust scavenging at 17PC suggests that the northern edge of the ITCZ was closer to that site, since at this time the source of dust to all three cores was most likely in the Northern Hemisphere. The hemispheric thermal gradient (Supplementary Note 2) supports this interpretation, as it shows rapid, deglacial Antarctic warming at a time when North Atlantic foraminifera assemblages and alkenone unsaturation indices record cold sea-surface temperatures (cf. Fig. 2e, f). This millennial-scale response of the ITCZ to changes in the hemispheric thermal gradient is consistent with models that predict ITCZ shifts in response to North Atlantic Heinrich stadials^4^.

Planktonic oxygen isotope data from the three sites are consistent with our inference of a southward-shifted ITCZ during the deglaciation. All three cores show a shift towards values indicating warmer, fresher conditions, but the change is largest at 17PC where a southward-shifted ITCZ may have warmed that site and/or more ITCZ precipitation may have freshened surface waters. In contrast, sites 31BB and 37BB experienced more muted freshening and warming over this time period as might be expected if the δ^18^O signal from decreasing precipitation opposed the effect of warming deglacial temperatures and decreasing ice volume.

The small lag in the onset of dust flux increases in cores 31BB and 17PC relative to the onset of HS11 is likely an artefact of uncertainty in our age models (see Methods) and we therefore do not interpret this offset as a climate system lag. Interestingly, core 37BB does not appear to show an increase in dust flux coincident with the records from our other two cores. We suggest two possible reasons for this observation. First, core 37BB may be seeing relatively little dust deposition during this otherwise high-dust-abundance time period. Because it would lie significantly (∼7°) north of the ITCZ at this time, it is possible that dust is inefficiently scavenged from the air column above the site. Alternatively, it could be that core 37BB experienced a significant increase in dust flux during HS11, an increase that has been subsequently obscured due to relatively lower accumulation rates and the effects of bioturbation (Supplementary Note 1).

Dust levels remain above the interglacial baseline in the southernmost core until ∼126 ka, which could indicate that the ITCZ was in its southernmost position until that time. However, we suggest that it is more likely that the ITCZ actually began to shift northwards around 129 ka when dust fluxes dropped below pre-stadial values, coincident with interglacial warming with stronger warming in the Northern Hemisphere. Our data display a slightly longer duration of HS11, probably due to the smoothing effects of bioturbation. This would cause HS11 to appear longer in our dust records than reconstructed from other proxies such as speleothems^27^, which have better age control and higher resolution but do not directly record dust fluxes or shifts in the ITCZ.

During MIS5e, all three cores record a decrease in dust flux to minimum values between 123 and 119 ka. On the basis of near-identical dust flux data for the two more northerly cores 37BB and 31BB, we suggest that at MIS5e the ITCZ was northwards of 7° N and that it was not influencing any of our sites during the peak interglacial. Planktonic oxygen isotope values which are indistinguishable at the three sites during MIS5e, are consistent with this argument, as an ITCZ located north of all three cores would be unlikely to differentially influence surface hydrography or temperature at any of the three sites. It is notable that after the ITCZ retreats northward during MIS5e that the δ^18^O at site 17PC does not show a 0.8‰ enrichment relative to cores 37BB and 31BB, as it did during the peak of MIS6. As suggested previously by the work on a larger set of ML1208 δ^18^O records^19^, this could be due to differences in the salinity of water carried by the North Equatorial Counter Current during glacial versus interglacial times. If the North Equatorial Counter Current carried fresher water from the western Pacific warm pool during MIS6 than during MIS5, a larger δ^18^O gradient would be predicted between equatorial and more northerly cores.

Our interpretation of an ITCZ located north of 7° N during MIS5e is consistent with a strong hemispheric thermal gradient, with the North Atlantic experiencing peak warmth at this time. At the end of MIS5e (∼119 ka) interglacial temperatures subsided in both hemispheres and dust fluxes began to rise, but the differential rate of cooling appears to have been small enough that the ITCZ was not displaced southward of 37BB, since 37BB and 31BB show comparable dust fluxes to the end of our record at 110 ka.

Only one other record of ITCZ movement exists for the penultimate deglaciation, a record of variations in the Al/Ti ratio from the Cariaco Basin^28^. The Cariaco Basin records ITCZ-driven changes in terrestrial runoff with higher (lower) ratios indicating more (less) runoff and therefore a relatively more northerly (southerly) ITCZ (Fig. 3b). Given that the Cariaco record is interpreted as recording relative movement of the terrestrial ITCZ, the agreement between our data sets is noteworthy and is consistent with the idea that ITCZ movement is likely coherent across large distances^2^ even on short timescales.

Model reconstructions of past ITCZ migrations have been challenging in part because of the sensitivity of the ITCZ to small changes in cloud feedbacks and energy balances^2^. Here we have shown that palaeo reconstructions can constrain the magnitude of even abrupt ITCZ movement, demonstrating the potential to relate ITCZ changes to the magnitude of thermal forcing and to investigate thermal and hydrological components of other climate change events. Our results provide evidence that an abrupt, millennial climate oscillation was a prominent feature of the penultimate deglaciation, both at high and low latitudes. During HS11, in concert with North Atlantic cooling^29^, ice rafting^25^, increases in northern hemisphere dust abundance, and a shift in the interhemispheric thermal gradient^29^,^30^, the ITCZ migrated southwards by at least 4° relative to MIS6, and 6.5° south of its present latitude. This shift likely had significant consequences for global atmospheric circulation^10^ and could thus have contributed to tipping the climate system over the threshold for deglaciation.

## Methods

### Age model development and uncertainty

Age–depth relationships for cores ML1208-37BB, 31BB and 17PC were developed on the basis of planktonic oxygen isotope stratigraphies^19^ tied to the LR04 benthic stack^31^, with the assistance of a modified Monte-Carlo-enabled cross-correlation maximization scheme and random walk algorithm (MonteXCM)^32^. A record of coeval planktonic and benthic oxygen isotopes values was previously reconstructed in the Eastern Equatorial Pacific^33^, which showed zero offset between the timing of deglaciation in the two stratigraphies. This observation gives us confidence that our use of planktonic oxygen isotope stratigraphies is not introducing a significant systematic bias into the timing of our events relative to data from the ice cores.

The use of the random walk algorithm in MonteXCM allows us to quantify age uncertainty at each data point. On average, over the interval studied, 2 s.d. uncertainties are 1.5, 1.5 and 1.7 ka for cores 37BB, 31BB and 17PC, respectively. Specifically, for the HS11 interval (129–136 ka), uncertainties are ∼1.3, 1.4 and 1.4 ka for the same three cores. The magnitude of these uncertainties means that it is probable that the small offsets in event timing observed between our three sediment cores are artefacts of the age model and do not represent true differences in the timing of the reconstructed dust flux changes or ITCZ shifts. However, our age constraints are sufficient to indicate that it is unlikely that these events are temporally disconnected, and a lag between the onset of HS11 and our inferred initiation of tropical changes is not inconsistent with the interpretations we present.

### Dust flux reconstruction

We reconstruct dust fluxes through the quantification of ^230^Th_xs,0_-normalized ^232^Th fluxes in marine sediments, which act as a proxy for dust flux^13^,^20^. Terrestrial sediments contain ^232^Th at a mean concentration of 10.7 p.p.m., which varies 1 p.p.m. or less over a range of dust provenance^13^. A more recent summary of ^232^Th concentrations in dust source areas found evidence that the fine fraction (<5 μm) of dust samples from a range of provenance had ^232^Th concentrations closer to 14±1 p.p.m. (ref. 34). We note that changing the ^232^Th concentration used to reconstruct dust fluxes would decrease the absolute magnitude of the dust fluxes reconstructed at our study sites but does not alter our interpretations. As our core locations are a great distance from continental sources of lithogenic material, we expect ^232^Th at our sites to be delivered predominately by aeolian processes. Indeed, grain size analyses show that the average size of lithogenic sediments at ∼1° N 131° W is 3.1 μm (ref. 35), unlikely to have been derived from hemipelagic sedimentation. Here, as in other studies^13^,^36^, dust fluxes based on ^232^Th are evaluated by normalization to ^230^Th_xs,0_ (ref. 37).

### Uranium and thorium geochemistry

Sediment samples from ML1208-37BB, 31BB and 17PC were analysed for uranium and thorium isotopes by isotope dilution and inductively coupled plasma mass spectrometry (Element XR, ICP-MS) at the Lamont–Doherty Earth Observatory of Columbia University. Between 100 and 200 mg of sediment per sample was spiked with ^236^U and ^229^Th before sediment dissolution and digestion with HNO_3_, HClO_4_ and HF (ref. 38). Anion-exchange column chemistry was also used to isolate the U/Th fraction^39^.

Samples were run with an external in-house standard (mirroring the physical and geochemical properties of our samples) to determine measurement reproducibility, yielding relative s.d.s of 0.74% for ^238^U, 1.94% for ^230^Th and 0.87% for ^232^Th. Background contamination was evaluated in each run via blanks, which were spiked and digested, but contained no sediment. Results show ^238^U blanks (0.4 ng), ^230^Th blanks (0.009 pg) and ^232^Th blanks (1.1 ng) all of which are <1% of even the lowest sample values. Errors reported in Fig. 2 represent 2 s.d. uncertainty estimates unique to each sample, including the propagated error due to uncertainty in counting statistics, mass bias corrections (from measurements of a natural ^238^U/^235^U standard), counting gain corrections, spike measurements, the lithogenic fraction (0.7±0.1)^40^ and a 1% age uncertainty. The average s.d. of replicate dust flux values is ∼6.4% of sample values. Samples for which dust flux values differed from a same-depth replicate, or an average of four nearest-neighbour data points by more than 4 s.d., have been excluded from the data set. The number of data points excluded using this method is <5% of the total number of data points reported.

### Data archiving

Line Islands dust flux data are archived at the National Oceanic and Atmospheric Administration National Centers for Environmental Information (NCEI) database and are also available as a supplement to this manuscript (Supplementary Data 1).

## Additional information

**How to cite this article:** Jacobel, A. W. *et al*. Large deglacial shifts of the pacific intertropical convergence zone. *Nat. Commun.* 7:10449 doi: 10.1038/ncomms10449 (2016).

## Supplementary Material

Supplementary Information Supplementary Figure 1, Supplementary Notes 1-2 and Supplementary References

Supplementary Data 1 Dust fluxes and associated 2 s.d. errors for sediment samples taken from cores ML1208-37BB, 31BB and 17PC. Values derived from ^230^Th_xs,0_-normalised ^232^Th concentrations.

## Figures and Tables

**Figure 1:**
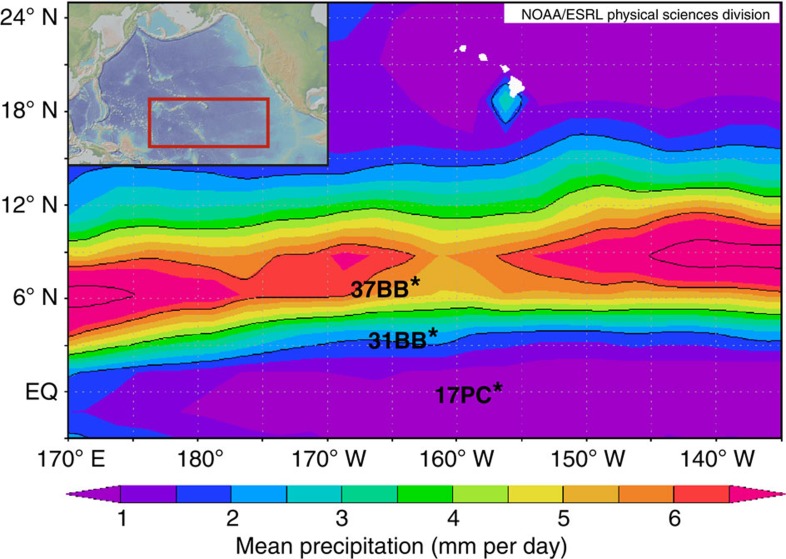
Map of study area. ML1208 core sites 37BB, 31BB and 17PC (black stars) are shown relative to Hawaii (white islands), and the modern mean annual position of the ITCZ, illustrated by the maximum mean annual precipitation in mm per day (coloured contours). Inset shows the location of this map (red rectangle) in the broader context of the Pacific Ocean. Basemap generated using the global precipitation climatology product (GPCP) version 2.2 from National Oceanic and Atmospheric Administration's (NOAA's) Physical Sciences Division, Earth System Research Laboratory. Inset generated in GeoMapApp.
