# Supplementary material for: Large deglacial shifts of the Pacific Intertropical Convergence Zone
Source: Nat Commun. 2016 Jan 22;7:10449. doi: 10.1038/ncomms10449 (PMC4735863; doi:10.1038/ncomms10449)
Supplement: Supplementary Information — Supplementary Figure 1, Supplementary Notes 1-2 and Supplementary References [file ncomms10449-s1.pdf]

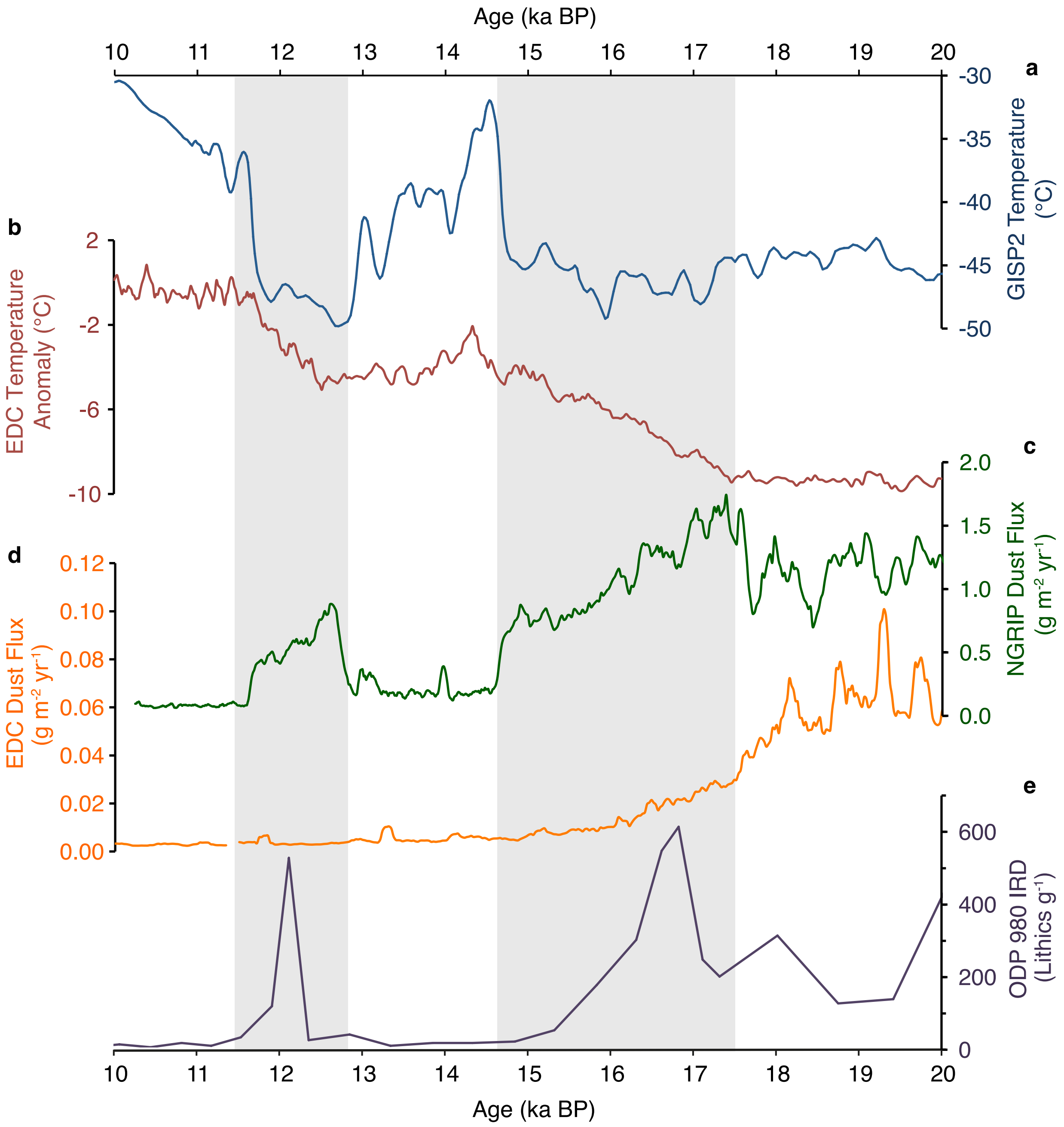

Supplementary Figure 1. Termination I data comparison. Greenland GRIP (72°35′N 37°38′W) temperature record (3pt smoothed)[1] **(a)**. Antarctic EDC (75°06'S, 123° 21'E) temperature anomaly (relative to the last 1,000 yrs) on AICC2012 chronology (5pt smoothed)[2,3] **(b)**. Greenland NGRIP (75°05'N, 42°19'W) dust flux on AICC2012 chronology (5pt

smoothed)[4,3] **(c)**. Antarctic EDC dust flux on AICC2012 chronology (5pt smoothed)[5,3] **(d)**. North Atlantic ODP 980 (55°29'N, 14°42'W) ice rafted debris (IRD) abundance[6] **(e)**. Grey shading indicates the Younger Dryas and Heinrich Stadial 1[7].

Supplementary Note 1

Effect of Bioturbation on ML1208 Dust Flux Records

Open ocean sediment accumulation rates vary widely in response to local geography, bathymetry, bottom current activity, surface productivity, water column dissolution, and many other variables. Other variables held constant, higher sedimentation rate regions are typically considered more ideal for paleoceanographic reconstructions as cores can be sampled at higher resolution with lower amounts of signal disturbance due to bioturbation.

Benthic and infaunal organisms moving along and burrowing into the ocean floor cause stochastic mixing of surrounding sediments, known as bioturbation. The cumulative effect of these actions is that, on a small scale, individual sediment constituents do not necessarily adhere strictly to laws of chronographic superposition. The effect of bioturbation on a sediment core can be modeled as a diffusive process[8], whereby proxy values at a given depth represent a combination of values at, above and also below that depth[9]. When the amount of bioturbation is the same, the smoothing effect is greater on cores with lower sedimentation rates. Several studies have attempted to quantify the length scale of bioturbation but the results vary greatly depending on the measurement method, season of measurement, faunal assemblage present[10] and organic content of the sediment[9]. Despite these challenges, considering the effects of bioturbation can be important for interpreting climate data as the precise timing, character and even frequency[11] of events can be altered by this post-depositional processes.

Bioturbation is likely an important process in sediment cores from the productive Line Islands region and should be considered when interpreting the changes in dust flux values between our three sediment cores. Given the geographic proximity and comparable water depths of the three ML1208 sites in this study, we make the assumption that these cores experienced similar rates of bioturbation. Thus, for the same magnitude of proxy change in all three cores (for example the dust flux increase during HS11), the core with the highest sedimentation rate (31BB) would be expected to retain a larger event signal than a lower sedimentation rate core (such as 37BB or 17PC). This effect of bioturbation is particularly important for our interpretations at HS11 because the core that records the largest dust flux excursion during HS11 (17PC) is not the highest sedimentation rate core. This suggests that the magnitude of the event at site 17PC was truly larger than at site 31BB (which has a higher sedimentation rate by ~1.0 cm ka^{-1}), supporting our inference that the ITCZ was likely closer to site 17PC during HS11.

Supplementary Note 2

Interpretation of the Hemispheric Thermal Gradient

The ITCZ has been shown to vary its position in response to seasonal shifts in the temperature contrast between hemispheres and on longer timescales in response to changes in solar forcing[12]. Typically, the ITCZ is positioned at the latitude where the atmospheric energy flux changes sign from positive to negative[12], that is where one branch of the Hadley circulation transports energy southwards and the other transports energy northwards. When one hemisphere is warmer, heat is transported from that hemisphere into the cooler hemisphere with the latitude of "zero" heat flux located in the warmer hemisphere. The larger the temperature difference between the hemispheres, the higher the latitude of the thermal equator. Specifically in this

study, this would indicate that the warmer the southern hemisphere relative to the northern hemisphere, the further southwards the ITCZ.

Other than estimates of the position of the ITCZ, no paleo record exists which reveals the integrated thermal relationship between Earth's hemispheres. However, comparing temperature records from each of the poles allows us to roughly approximate this balance through time. Ideally, this comparison would be made using the same proxy at comparable northern and southern hemisphere latitudes. Ice core records would be preferable since the conventional interpretation of deuterium isotopes (δD_{ice}) is directly related to surface temperature[2]. For the southern hemisphere we use a record of δD_{ice} from the Antarctic EPICA Dome C ice core[2]. Unfortunately, Greenland's ice core records do not currently extend to the penultimate deglaciation (Termination II) so another proxy is required. Here, we use two proxies, a North Atlantic reconstruction of sea surface temperatures (SSTs) using the modern analogue technique (MAT)[13] and another reconstruction of SSTs from the Iberian Margin derived from alkenones[14]. These are imperfect proxies for comparison to Antarctic surface temperatures but nonetheless provide a useful first-order constraint on the relative magnitudes and rates of warming in the two hemispheres. A more nuanced quantification of the magnitude of thermal forcing expressed during the penultimate deglaciation and its relationship to ITCZ position/shifts would be valuable, particularly for modeling efforts, but is beyond the scope of this paper. Despite their spatio-temporal limitations, the records from the northern and southern hemispheres presented in Fig. 2 panels e and f, are in agreement with our ITCZ interpretations, as the sense of movement is entirely consistent with the large-scale changes in the thermal balance between hemispheres.

Our argument for the out of phase deglacial temperature variations in the North Atlantic and southern hemisphere during MIS6-5 is strengthened by the similar pattern associated with

Termination 1 (Supp. Fig. 1 panel a). During both deglaciations Antarctic temperatures began to rise while the North Atlantic and Greenland remained cold (Supplementary Figure 1 panels a and b, and Fig. 2 panels e and f). An increase in seasonal sea ice cover likely further decreased winter temperatures and may have led to iceberg rafting which delivered IRD (Fig 2 panel d and Supplementary Figure 2 panel e) and freshwater to the North Atlantic[15]. Other authors have previously observed these and other parallels between the last two deglaciations[16] so while coincident behavior of interhemispheric temperature patterns is not a new finding, the millennial scale influence of the thermal balance on ITCZ position during Termination II is a striking observation.

Supplementary References

1. Alley, R. B. The Younger Dryas cold interval as viewed from central Greenland. *Quaternary Science Reviews* **19**, 213–226 (2000).
2. Jouzel, J. *et al.* Orbital and Millennial Antarctic Climate Variability over the Past 800,000 Years. *Science* **317**, 793–796 (2007).
3. Veres, D., Bazin, L. & Landais, A. The Antarctic ice core chronology (AICC2012): an optimized multi-parameter and multi-site dating approach for the last 120 thousand years. *Climate of the Past* **9**, 1733–1748 (2013).
4. Ruth, U. *et al.* Ice core evidence for a very tight link between North Atlantic and east Asian glacial climate. *Geophysical Research Letters* **34**, L03706 (2007).
5. Lambert, F. *et al.* Dust-climate couplings over the past 800,000 years from the EPICA Dome C ice core. *Nature* **452**, 616–619 (2008).
6. McManus, J. F., Oppo, D. W. & Cullen, J. L. A 0.5-million-year record of millennial-scale climate variability in the North Atlantic. *Science* **283**, 971–975 (1999).
7. Wang, Y. J. *et al.* A High-Resolution Absolute-Dated Late Pleistocene Monsoon Record from Hulu Cave, China. *Science* **294**, 2345–2348 (2001).
8. Boudreau, B. P. Is burial velocity a master parameter for bioturbation? *Geochimica et Cosmochimica Acta* **58**, 1243–1249 (1994).
9. Ruddiman, W. F. & Glover, L. K. Vertical Mixing of Ice-Rafted Volcanic Ash in North Atlantic Sediments. *Geological Society of America Bulletin* **83**, 2817–2835 (1972).
10. Teal, L. R., Bulling, M. T., Parker, E. R. & Solan, M. Global patterns of bioturbation intensity and mixed depth of marine soft sediments. *Aquatic Biology* **2**, 207–218 (2008).
11. Goreau, T. J. Frequency sensitivity of the deep-sea climatic record. (1980).
12. Schneider, T., Bischoff, T. & Haug, G. H. Migrations and dynamics of the intertropical convergence zone. *Nature* **513**, 45–53 (2014).

13. Oppo, D. W., McManus, J. F. & Cullen, J. L. Evolution and demise of the Last Interglacial warmth in the subpolar North Atlantic. *Quaternary Science Reviews* **25**, 3268–3277 (2006).
14. Pailler, D. & Bard, E. High frequency palaeoceanographic changes during the past 140 000 yr recorded by the organic matter in sediments of the Iberian Margin. *Palaeogeography, Palaeoclimatology, Palaeoecology* **181**, 431–452 (2002).
15. Barker, S. *et al.* Icebergs not the trigger for North Atlantic cold events. *Nature* **520**, 333–336 (2015).
16. Cheng, H. *et al.* Ice Age Terminations. *Science* **326**, 248–252 (2009).
